# A Review on Registration Techniques for Cardiac Computed Tomography and Ultrasound Images

**DOI:** 10.3390/bioengineering12121351

**Published:** 2025-12-11

**Authors:** Zongyang Li, Huijing He, Qi Wang, Luyu Li, Hongjian Gao, Jiehui Li

**Affiliations:** 1 Department of Biomedical Engineering, College of Chemistry and Life Science, Beijing University of Technology, Beijing 100124, China; 1983732493@emails.bjut.edu.cn (Z.L.); hhj975445@emails.bjut.edu.cn (H.H.); wangqi23@emails.bjut.edu.cn (Q.W.); antonia@emails.bjut.edu.cn (L.L.); 2 State Key Laboratory of Cardiovascular Disease, Department of Cardiac Surgery, National Center for Cardiovascular Diseases, Fuwai Hospital, Chinese Academy of Medical Sciences, Peking Union Medical College, Beijing 100037, China

**Keywords:** image registration, deep learning, deformation field

## Abstract

With the rapid development of medical imaging technology, the early diagnosis and treatment of heart disease have been significantly improved. Cardiac CT (Computed Tomography) and ultrasound images are often used in combination to provide more comprehensive information on cardiac structure and function due to their respective advantages and limitations. However, due to the significant differences in imaging principles, resolutions, and viewing angles between these two imaging modalities, how to effectively register cardiac CT and ultrasound images has become an important research topic in imaging and clinical applications. This article summarizes the research progress of cardiac CT and ultrasound image registration, and analyzes the existing registration methods and their advantages and disadvantages. Firstly, this article summarizes traditional registration methods based on image intensity, feature points, and regions, and explores the application of rigid and non-rigid registration algorithms. Secondly, in view of common challenges in cardiac CT and ultrasound image registration, such as image noise, deformation, and differences in imaging time, this article discusses the recent advances in multimodal registration technology in cardiac imaging and forecasts the potential of deep learning methods in registration. In addition, this article also evaluates the application effects and limitations of these methods in clinical practice, and finally looks forward to the future development direction of cardiac image registration technology, especially its potential applications in personalized medicine and real-time monitoring. Through a comprehensive review of the current research status of cardiac CT and ultrasound image registration, this article provides a systematic theoretical framework for researchers in related fields and provides a reference for future technological breakthroughs and clinical translation.

## 1. Introduction

Cardiovascular disease remains one of the leading causes of mortality worldwide. Early diagnosis and precise treatment are essential for improving patient survival and quality of life. Traditional diagnostic approaches largely rely on clinical symptoms, physical examination, and basic biochemical markers. However, these methods often lack the sensitivity and specificity required to detect early-stage pathological changes [1].

With the rapid advancement of imaging technologies, cardiac imaging has become a cornerstone in the diagnosis and assessment of cardiovascular diseases. It provides clinicians with detailed anatomical and functional information about the heart, facilitating comprehensive evaluation of hemodynamics, coronary artery stenosis, myocardial wall thickness and motion, as well as myocardial ischemia [2]. In particular, cardiac imaging plays a pivotal role in the diagnosis and management of conditions such as coronary artery disease, heart failure, valvular heart disease, and congenital heart anomalies, offering high-resolution visual data to support individualized clinical decision-making.

In recent years, the development of imaging techniques such as CT, MRI (Magnetic Resonance Imaging), and ultrasound has provided strong support for cardiac function assessment, cardiovascular interventional planning, and postoperative follow-up. Cardiac CT, with its sub-millimeter spatial resolution, offers a critical basis for the early diagnosis of atherosclerosis. In contrast, echocardiography, with its advantage of real-time dynamic imaging, has become the preferred modality for evaluating ventricular synchrony and hemodynamics [3]. To maximize the diagnostic value of these two modalities, it is essential to establish an accurate cross-modality image registration framework. By spatially aligning the anatomical precision of CT with the functional kinetic characteristics derived from ultrasound, this technique effectively overcomes the inherent limitations of single-modality imaging. Such multimodal registration enables fused imaging for preoperative 3D surgical planning and facilitates real-time intraoperative comparison between ultrasound and preoperative CT models. This significantly improves the precision of complex procedures such as mitral valve repair and left atrial appendage occlusion. With the continuous improvement of multimodal fusion systems, cardiac imaging is evolving from a diagnostic adjunct into a core foundation for individualized therapeutic guidance.

In summary, cardiac imaging plays an indispensable role in early screening, quantitative assessment, and therapeutic planning for cardiovascular diseases. It provides robust technological support for precision medicine and personalized treatment strategies. As imaging technologies continue to evolve, cardiac imaging is poised to play an increasingly prominent role in disease prevention, health management, and clinical decision-making.

This review aims to systematically summarize recent advances in image registration between CT and echocardiography, with a particular focus on the technical principles and clinical applications of both traditional registration frameworks and deep learning-based approaches. By comparatively analyzing the two paradigms in terms of feature extraction accuracy, registration efficiency, and clinical performance, this study seeks to elucidate current methodological bottlenecks and propose directions for future optimization.

### 1.1. Traditional Registration Framework

**Figure 1:**
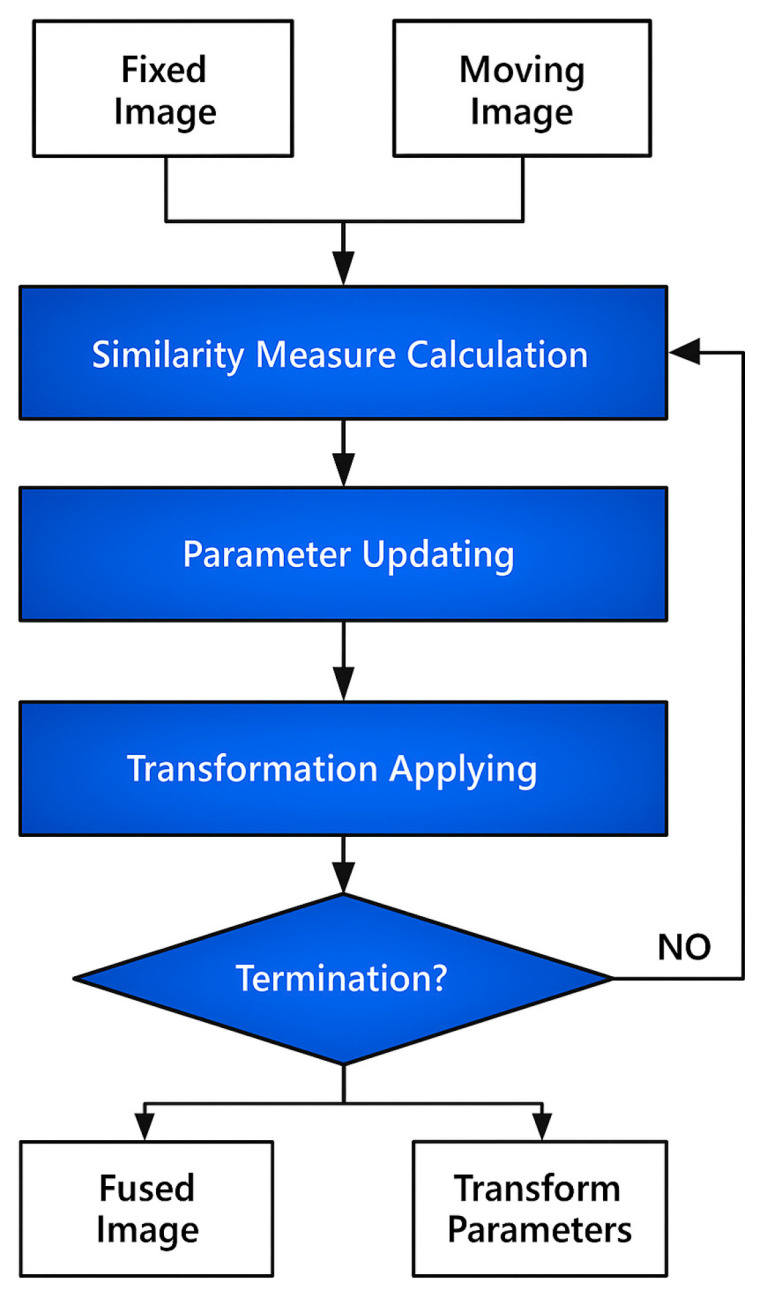
Traditional Image Registration Process.

Image registration refers to the process of spatially aligning two or more medical images, with the goal of eliminating geometric discrepancies between them and ensuring consistent spatial positioning and structural information within a common coordinate system [4]. Figure 1 shows that the image registration is to find the optimal spatial transformation of one image to another through optimization algorithms, so that the two images achieve the greatest degree of consistency in anatomical structure or functional information. Depending on the nature of the transformation applied, registration techniques can be broadly categorized into rigid, affine, and non-rigid registration methods [5]. Rigid registration accounts for rotation and translation, while affine registration further incorporates scaling and shearing transformations. Non-rigid registration, on the other hand, allows for more complex local deformations, making it particularly suitable for aligning anatomical structures that may change shape over time or vary across individuals.

Moreover, medical image registration techniques can be classified into feature-based and intensity-based approaches [6]. Feature-based methods rely on the extraction and alignment of salient anatomical landmarks or contours, whereas intensity-based methods directly utilize voxel intensity values and employ similarity metrics–such as mutual information or cross-correlation–to guide the alignment process.

### 1.2. Deep Learning-Based Registration Framework

**Figure 2:**
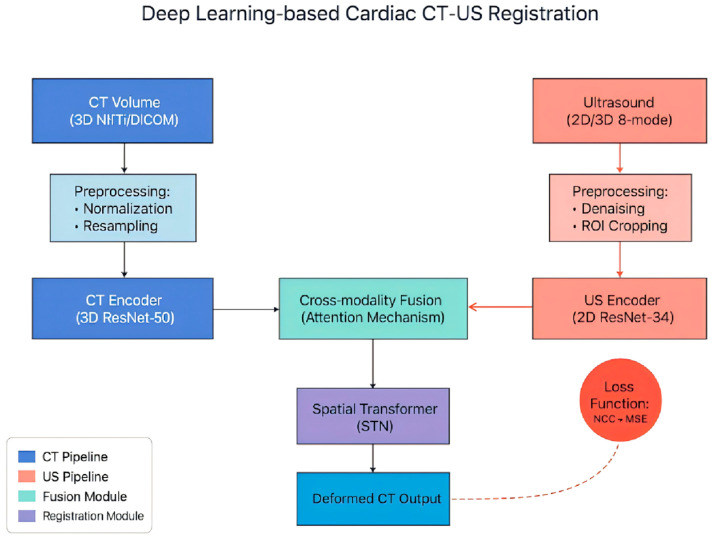
Deep Learning-based Registration Process. The light blue part is the pretreatment process of CT image, the light red part is the pretreatment process of ultrasound image, and the dark red part is the loss function to guide the optimization of registration model.

The deep learning-based registration of cardiac CT and ultrasound images typically involves three key stages: feature extraction, spatial transformation, and alignment optimization. V. Ajantha Devi investigated a CNN-based approach for cardiac CT–ultrasound multimodal image registration, targeting the challenge of achieving anatomically precise alignment to integrate complementary structural and functional information for cardiovascular diagnosis [7]. As illustrated in Figure 2, the CT–US registration pipeline is adapted from the original study, and its major components follow the authors’ design. A dual-branch CNN encoder is employed to extract high-resolution anatomical features from CT images, as well as dynamic functional cues from ultrasound. The workflow includes preprocessing of 3D CT and 2D/3D ultrasound inputs, dual-branch feature extraction, attention-based feature fusion, and spatial-transformer-network (STN)-driven registration. Optimization is guided by an NCC–MSE hybrid loss, ensuring alignment accuracy and producing the final deformed CT output.

Chen et al. proposed the use of a cross-modal attention mechanism to compute a similarity matrix between the two modalities in the feature space, thereby generating an initial deformation field. A 3D U-Net architecture is then employed to fuse multi-scale features and enhance local detail alignment [8]. In the preprocessing stage, CT images are typically thresholded to remove bony artifacts and suppress speckle noise in ultrasound images. An initial coarse alignment is often achieved through elastic registration, which helps reduce the optimization burden on subsequent deep learning models.

In terms of network architecture, most state-of-the-art methods adopt a cascaded framework. Lei et al. developed the Deformable Vision Transformer [9], which leverages self-attention mechanisms to capture temporal consistency across cardiac cycles. This model incorporates a cycle-consistency loss to constrain the deformation trajectories between systolic and diastolic phases, ensuring temporal coherence.

To address the substantial modality gap between CT and ultrasound, some studies have introduced adversarial training strategies. For example, Yao’s group designed a modality-invariant generator that utilizes a gradient reversal layer to suppress domain-specific features [10]. Their method achieved a Dice similarity coefficient of 0.89 ± 0.03 on the MICCAI public dataset, representing a 12% improvement over traditional approaches.

In the post-processing stage, biomechanical constraints are often incorporated. Finite element analysis is employed to validate the physiological plausibility of the deformation field, helping to prevent anatomical distortions caused by overfitting.

## 2. Method

### 2.1. Search Strategy

To conform to PRISMA guidelines, a structured and transparent search strategy was implemented. A comprehensive literature search was conducted across four major databases—PubMed, IEEE Xplore, Scopus, and Google Scholar. The search strategy combined controlled vocabulary and free-text terms related to cardiac imaging and multimodal registration. Keywords included: “cardiac CT,” “ultrasound,” “image registration,” “multimodal registration,” “deep learning,” “intensity-based registration,” and “feature-based registration.” Boolean operators (AND/OR) were used to systematically combine search terms and expand the coverage of relevant studies.

In the identification phase, all retrieved records were imported into a reference management system, and duplicates were removed. During the screening phase, titles and abstracts were evaluated against predefined inclusion and exclusion criteria. Eligible studies were those (1) published within the last twenty years, (2) peer-reviewed journal articles or international conference papers, and (3) directly addressing cardiac CT–ultrasound image registration. Studies focusing on non-cardiac or unrelated registration tasks, non-human data, or lacking methodological detail were excluded. Full-text assessment was subsequently performed to confirm eligibility. The final set of studies included in the review represents those meeting all criteria for relevance, methodological adequacy, and scientific quality.

### 2.2. Selection Criteria

We applied strict inclusion criteria to ensure that only studies directly relevant to the registration of cardiac CT and ultrasound images were considered. Specifically, the following categories of literature were excluded:

1. Studies focusing solely on single-modality image registration, such as those involving only CT or only ultrasound image alignment.

2. Studies in which the target anatomy was not the heart, including those addressing image registration of other anatomical regions such as the brain, liver, or other non-cardiac structures.

### 2.3. Screening Process

During the literature screening process, all retrieved records were first imported into a reference management tool for deduplication to remove duplicate entries. The remaining studies underwent an initial screening based on titles and abstracts to identify potentially relevant works related to cardiac CT and ultrasound image registration. Subsequently, full-text articles were assessed for eligibility according to predefined inclusion and exclusion criteria, focusing on methodological rigor, relevance to multimodal image registration, and availability of quantitative results. Studies with insufficient methodological detail, non-cardiac focus, or irrelevant imaging modalities were excluded. Finally, 50 representative studies were included in the qualitative synthesis based on their methodological innovation, research quality, and potential clinical applicability.

## 3. Results

### 3.1. Overview of Intensity-Based Registration Techniques

Intensity-based image registration methods establish spatial correspondences by directly leveraging image intensity values, and they play a significant role in the cardiac CT-ultrasound registration. Table 1 summarizes the representative studies on intensity-based medical image registration methods in cardiac CT and ultrasound imaging in recent years. This table conducts a systematic comparative analysis of registration modalities, transformation models, and registration techniques. Traditional approaches often rely on optimizing similarity metrics such as mutual information (MI) and normalized mutual information (NMI), in conjunction with affine or elastic transformation models. These models are typically optimized using iterative algorithms such as gradient descent or genetic algorithms to estimate the optimal registration parameters. To address the challenges posed by multimodal cardiac imaging, preprocessing steps such as noise suppression and structural enhancement are commonly introduced to improve registration robustness, while multi-resolution strategies are employed to mitigate the effects of local minima during optimization.

However, the speckle noise, viewpoint dependency, and significant intensity distribution differences inherent in ultrasound images often render traditional mono-modal similarity metrics ineffective. To overcome these limitations, advanced techniques such as Local Mutual Information (LMI) and Modality Independent Neighborhood Descriptor (MIND) have been developed, which enhance cross-modal similarity estimation by incorporating local statistical features. Given the dynamic nature of cardiac motion, four-dimensional spatiotemporal registration approaches have also emerged, incorporating periodic motion models-such as B-spline deformation fields-to simultaneously correct for temporal misalignment.

Most methods achieve temporal phase matching through electrocardiogram (ECG) gating, selecting CT and ultrasound images from the same cardiac cycle phase, and utilize interpolation techniques to enhance temporal consistency. In terms of spatial registration, it leverages the complementary characteristics of high-resolution anatomical structures from CT and dynamic functional information from ultrasound, directly utilizing image gray-level information and normalized mutual information (NMI) as a similarity measure, combined with rigid or affine transformation models to optimize alignment. During the preprocessing stage, Speckle Reducing Anisotropic Diffusion (SRAD) [16] is often introduced to reduce ultrasound noise, while anisotropic diffusion is applied to CT data to maintain edge smoothness. Optimization algorithms such as pattern search and gradient descent are used to iteratively solve for optimal transformation parameters. Some studies improve accuracy through multi-plane registration, such as orthogonal ultrasound cross-sections, effectively reducing single-view bias.

Recent efforts have focused on improving the robustness and clinical adaptability of registration frameworks. Khalil et al. proposed an automated method without optical tracking, using NMI maximization to align cardiac CT and ultrasound, and demonstrated its feasibility in mitral valve navigation [17]. Dahman et al. developed a biplanar ultrasound–CT registration system combining mutual information and fiducial markers, reducing target registration error (TRE) from 2.54 mm to 1.7 mm [12]. Abiri et al. applied deformable registration to create a 4D cardiac motion model, enabling high-precision data for virtual reality surgical simulation [14]. These studies, validated on clinical data, address the challenges of non-rigid cardiac motion and real-time imaging, offering practical solutions for CT–ultrasound fusion in interventional procedures.

### 3.2. Overview of Feature-Based Registration Techniques

Due to significant differences in imaging principles and appearance between CT and ultrasound, intensity-based registration often performs poorly. Therefore, traditional methods focus on extracting modality-invariant features such as key points, anatomical landmarks, and structural contours. These features facilitate the establishment of spatial correspondence, demonstrating high robustness to intensity variations and excellent computational efficiency. Common techniques include manual annotations or automatic detectors like SIFT and Harris corners. Jørn Bersvendsen et al. proposed a fully automatic spatio-temporal registration method for 4D cardiac ultrasound, achieving temporal alignment via normalized cross-correlation curve matching and spatial registration via time-independent FBA using 3D SIFT features and RANSAC-based rigid transformation, without external ECG [18]. Despite modality gaps, their method achieved reliable alignment (NMI = 0.62 ± 0.08). Similarly, Nordenfur et al. used semi-automatic registration based on mitral valve centers and LVOT planes, refined with surface-based Iterative Closest Point (ICP), reducing key-point errors [19]. These results show that combining global anatomical features with local surface optimization is effective for CT–ultrasound registration.

To address challenges posed by cardiac motion and complex local tissue deformation, traditional methods have explored more flexible transformation models. Jacob et al. proposed a staged optimization framework that first applies global affine registration, followed by local non-rigid refinement [20]. Initial alignment is achieved using anatomical landmarks on a three-chamber view, with subsequent deformation modeled via thin-plate splines. Their approach demonstrated low root-mean-square error in CT–ultrasound registration, confirming the effectiveness of staged transformation modeling for handling anatomical variability. Additionally, Machado et al. proposed cDRAMMS, a deformable MR–US registration framework for brain shift correction. It uses multi-scale, multi-orientation texture attributes, correlation-based similarity metrics (aNCC, aCR), and a rigid–masking–deformable strategy to address FOV mismatch, achieving consistent landmark error reduction and the first region- and tumor-specific evaluation [21]. These studies suggest that even under significant temporal and spatial variation, feature-based traditional methods can maintain high robustness and accuracy.

Overall, traditional feature-based methods for cardiac CT–ultrasound registration have demonstrated strong performance. Techniques centered on key-point extraction, anatomical landmark detection, intensity-invariant similarity metrics, and staged rigid-to-nonrigid transformation strategies have all contributed to improved cross-modality alignment accuracy. Reported studies commonly achieve Dice similarity coefficients in the range of 0.85–0.90, with registration errors typically controlled within 1–5 mm. While deep learning approaches have rapidly advanced in recent years, conventional methods-based on handcrafted features and physically interpretable transformations-continue to offer reliability and transparency, which remain valuable in clinical applications. Table 2 presents a summary and comparison of the following feature-based medical image registration methods.

**Table 2:** Feature-Based Image Registration for Cardiac CT and Ultrasound images.

Reference	Method	Transform	Modality	TRE
Jørn et al. [18]	SIFT	Rigid	US-US	2.8 ± 0.6 mm
Nordenfur et al. [19]	ICP	Rigid	CT-US	-
Machado et al. [22]	3D SIFT	Non-rigid	US-US	1.53 ± 0.01 mm
Jacob et al. [20]	ICP	Non-rigid	CT-US	-
Liu et al. [23]	SURF	Rigid	CT-US	0.96 ± 0.17 mm

### 3.3. Deep Learning Based Registration Model: Supervised Learning Model

**Figure 3:**
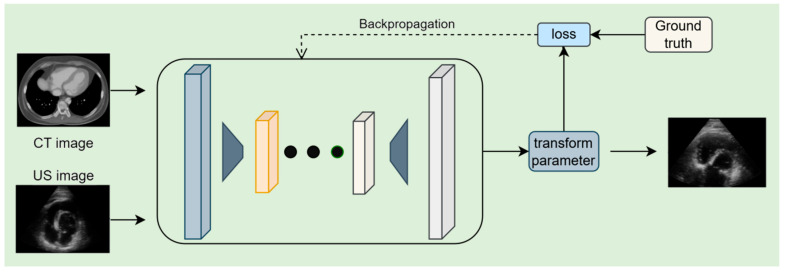
Cardiac CT–ultrasound registration model framework based on supervised learning for preoperative surgical planning.

Supervised learning models employ labeled training data to learn the mapping between input features and corresponding outputs, enabling the models to perform prediction or classification on unseen data. A typical supervised learning framework for cardiac CT–ultrasound registration, involving parameters such as input CT/US images, transform parameters for spatial alignment, and loss computed against ground truth via backpropagation to optimize registration, is illustrated in Figure 3. The methods mentioned below are shown in Table 3.

Supervised deep learning approaches have achieved notable progress in cardiac CT–ultrasound registration by integrating modality-specific information with anatomical knowledge. These methods learn explicit mappings between anatomical features and spatial transformations, substantially improving registration accuracy across modalities. Chen et al. pioneered a supervised framework based on anatomical landmarks, utilizing Mimics and 3-Matic software [4] to create alignment templates of key structures such as the mitral valve annulus. Their method enabled cross-modality mapping between CT and ultrasound through supervised feature matching, with animal studies confirming the effectiveness of the supervision signals in compensating for modality discrepancies. Building on this, Liu’s team introduced a two-stage supervised learning strategy [23]. In the first stage, landmark-guided similarity maximization was used to generate initial registered CT slices. In the second stage, a local supervised loss function based on SURF features was applied, enabling cascaded optimization. Phantom experiments demonstrated submillimeter accuracy with a mean registration error of 0.96 ± 0.17 mm, highlighting the advantage of supervised learning in multi-scale feature integration.

In dynamic cardiac registration scenarios, Gilbert et al., using the public ACDC dataset, developed a U-Net architecture incorporating anatomical constraints to enable precise multi-chamber cardiac segmentation [24]. Their supervised framework achieved a Dice coefficient of 0.833, underscoring the critical role of anatomical priors in improving cross-modality deep learning–based registration.

Supervised learning has also been widely applied in multi-label structure-guided registration. Nina et al. used a 3D U-Net to generate vessel labels, with vascular bifurcations enabling automatic rigid initialization [25]. They introduced a hybrid similarity metric combining MIND and NGF to drive B-spline non-rigid registration, achieving a deformation accuracy of 2.995 mm on 42 CT–3D ultrasound pairs. This approach effectively enhances cross-modality deformation modeling through semantic guidance.

To tackle cross-dimensional challenges, Toth et al. built a contour feature pyramid to efficiently select CT slices based on area and contour count, achieving real-time 2D ultrasound–3D CT registration through iterative optimization [26]. Li et al. proposed a decoupled training–inference framework [27]. Multi-scale features are extracted via a multi-branch U-Net during training, then re-parameterized into a lightweight single-path model for inference. With the Meta-ACON activation function [28], their method reached 0.08 mm displacement accuracy and 26 ms inference speed, demonstrating strong potential for real-time, cross-modal registration.

**Table 3:** Overview of supervised registration methods.

Reference	Network	Transform	Modality	Dice
Roelofs et al. [29]	3D Res-Net	Non-rigid	CT-CT	0.79 ± 0.23
Toth et al. [26]	CNN + MLP	Non-rigid	CT-X	-
Gilbert et al. [24]	CNN	Rigid	CT-US	-
Sokooti et al. [30]	Reg-Net	Non-rigid	CT-CT	0.78 ± 0.04
Eppenhof et al. [31]	3D U-Net	Non-rigid	CT-CT	0.798 ± 0.033

Supervised models are designed to learn the parameters or feature representations required for image registration from annotated data, aiming to enhance both the accuracy and efficiency of the registration process. During training, these models rely on ground-truth information to guide the network’s learning. Furthermore, they have demonstrated advantages over traditional methods or other comparative approaches in their respective experiments.

### 3.4. Deep Learning-Based Registration Model: Unsupervised Learning Model

**Figure 4:**
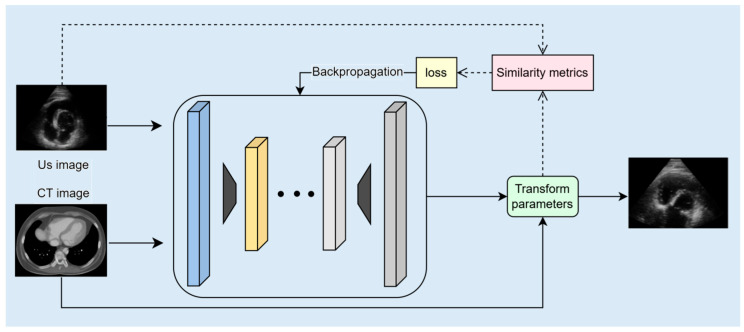
Cardiac CT–ultrasound registration model framework based on unsupervised learning for preoperative surgical planning.

Unsupervised learning models autonomously uncover the intrinsic structures and patterns within unlabeled data, facilitating the discovery of latent relationships and regularities. Unlike supervised methods, these models do not rely on manually annotated labels; instead, they employ techniques such as clustering, dimensionality reduction, and association rule mining to extract hidden features or data distributions. A typical unsupervised learning framework for CT–ultrasound registration, incorporating parameters such as input US and CT images, transform parameters for enabling spatial alignment, and similarity metrics for loss calculation via backpropagation to optimize the registration performance, is illustrated in Figure 4. An overview of unsupervised registration methods is illustrated in Table 4.

In recent years, unsupervised deep learning has shown strong potential in cardiac multimodal image registration, especially for dynamic cross-modal alignment between ultrasound and CT. Unsupervised approaches use self-supervised loss functions, making them well-suited for clinical scenarios with limited annotations. Khalil et al. proposed an early two-stage spatiotemporal framework, using ECG gating to select US frames aligned with CT phases [17]. A rigid registration model based on NMI enabled alignment of 2D US with 3D CT in the aortic valve short-axis view, achieving a Dice score of 0.81 ± 0.08 and demonstrating feasibility in anatomically sparse regions. To model non-rigid cardiac motion, Huang et al. introduced a B-spline Fourier (BSF) model that combines global motion via Fourier basis and local deformation via B-splines [32]. With consistency optimization, the method achieved 1.2 ± 0.8 mm accuracy on dynamic phantoms, laying a foundation for CT–US fusion. Building on this, Lei et al. developed a multi-scale deformable network (MS-DIRNet), with global and local branches to capture overall motion and fine anatomical differences [9]. An adversarial loss ensured deformation smoothness. On 4D-CT, the method reduced target registration error (TRE) by 52%, significantly improving temporal robustness.

With the integration of attention mechanisms and Transformer architectures, unsupervised registration methods have made significant progress in cross-modal feature disentanglement and structural alignment. Hasan et al. developed a Feedback Attention Module (FBA) that iteratively refines the displacement field using residual error maps [33]. Coupled with a spatiotemporal Transformer, the model captures long-range myocardial motion dependencies, achieving a Dice score of 0.881 in 3D ultrasound–CT registration of fetal hearts—a 10% improvement over VoxelMorph [5]. To meet real-time requirements in surgical navigation, Dahman et al. proposed a lightweight CNN combined with a Symmetric Log-Domain Diffeomorphic (SLDD) deformation model [34]. By maximizing mutual information and applying Jacobian regularization, their method enabled elastic registration of cardiac valves with a forward inference time under 100 ms, offering a 100-fold speedup over traditional DEMONS algorithms. These advancements demonstrate that unsupervised methods—through adaptive feature alignment, multi-scale fusion, and spatiotemporal consistency constraints—are overcoming longstanding accuracy and efficiency bottlenecks in US–CT registration. Such progress provides essential technical support for precise navigation in cardiac interventional procedures.

**Table 4:** Overview of unsupervised registration methods.

Reference	Network	Transform	Modality	Dice
Khalil et al. [17]	CNN + NMI	Rigid	CT-US	0.81 ± 0.08
Huang et al. [32]	U-Net + B-spline	Rigid	CT-US	-
Lei et al. [9]	MS-DIR-Net	Non-rigid	CT-CT	-
Hasan et al. [33]	FBA + DLIR	Non-rigid	CT-US	0.8 ± 0.07
Wiputra et al. [35]	HRF	Non-rigid	CT-US	0.812 ± 0.062
Dahman et al. [34]	CNN + STN	Non-rigid	CT-US	0.8 ± 0.02

### 3.5. Deep Learning-Based Registration Model: Weakly Supervised Learning Model

**Figure 5:**
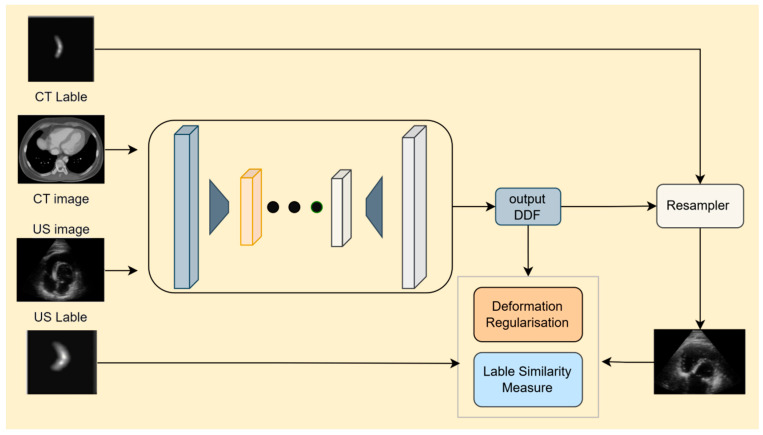
Cardiac CT–ultrasound registration model framework based on weakly supervised learning for preoperative surgical planning.

Weakly supervised image registration refers to techniques that guide the registration process using sparse annotations or indirect features, rather than relying on densely labeled data. This approach significantly reduces the dependence on extensive manual annotations and aligns more closely with practical clinical needs. The common weakly supervised registration processes, involving parameters like multimodal datasets (CT/US images and labels), dense deformation field (DDF) for spatial mapping, deformation regularization for smoothness, label similarity measures for alignment accuracy, and resampler parameters for spatial interpolation, are illustrated in Figure 5. An overview of the methods mentioned below is presented in Table 5, which displays the applicable models, transformation forms, and image types for each method.

Cardiac CT–ultrasound registration faces challenges such as inconsistent structural clarity, modality-dependent intensity differences, and motion artifacts from cardiac cycles. Weakly supervised learning offers significant advantages by leveraging anatomical priors and reducing the need for full annotations. Hu et al. proposed a framework based on cardiac valve plane geometry [36]. Key structures like the mitral and aortic valve annuli were automatically segmented, and geometric cues served as weak supervision. In CT–TEE fusion for atrial fibrillation patients, 92.68% of plane angle errors were below 5°, requiring only sparse landmark annotations. Similarly, Haishan et al. developed a classification–segmentation framework. A classifier estimated the ultrasound probe orientation, while segmentation refined the alignment using contours of cardiac chambers or valves. Weak supervision signals, such as vessel overlap, improved robustness in aligning dynamic ultrasound sequences.

The generalizability of weakly supervised cross-modal strategies has further advanced the clinical applicability of cardiac image registration. Ramalhinho et al. proposed a multi-label retrieval and Bayesian sequential optimization approach [37]. By generating a pre-computed library of CT-simulated vascular bifurcation planes, their method dynamically matches anatomical features in ultrasound sequences. This framework adapts well to cardiac settings by leveraging temporal consistency in chamber contraction-relaxation patterns, thereby reducing reliance on single-frame annotations. He et al. introduced a hybrid similarity metric that combines MIND-based contextual features constrained by segmentation masks with NGF-derived edge gradients [38]. This approach effectively addresses motion artifacts in cardiac ultrasound. By segmenting anatomical structures such as atria and ventricles to generate masks, the method enhances Non-rigid registration accuracy while minimizing manual intervention.

In summary, weakly supervised registration between cardiac CT and ultrasound is evolving from structure-guided models toward feature-level integration. By incorporating anatomical segmentation and indirect supervision signals, these methods reduce annotation costs while improving registration quality, offering robust technical support for clinical applications such as preoperative planning and image-guided interventions.

**Table 5:** Overview of weakly—supervised registration methods.

Reference	Network	Transform	Modality	Dice
Hu et al. [36]	CNN	Non-rigid	MRI-TRUS	0.73 ± 0.304
Fries et al. [39]	CNN + LSTM	Non-rigid	CT-MRI	-
Blendowski et al. [40]	Feat CNN	Non-rigid	CT-MRI	0.51 ± 0.02
Ferrante et al. [41]	LSSVM + MRF	Non-rigid	CT-MRI	0.788 ± 0.07
He et al. [38]	3D U-Net	Non-rigid	CT-US	0.799 ± 0.097

## 4. Discussion

This review explores the advanced techniques in cross-modal registration between cardiac CT and ultrasound images. Through a comparative analysis with traditional methods, we summarize that deep learning-based registration approaches demonstrate significant advantages in both accuracy and computational efficiency.

Supervised learning methods leverage annotated data (e.g., ground-truth deformation fields) to directly optimize registration networks, achieving high precision in aligning local anatomical features, such as coronary arteries and myocardial boundaries. However, the heavy reliance on large-scale, high-quality labeled datasets presents a major bottleneck, as the annotation process in medical imaging is both costly and dependent on expert knowledge, thus limiting clinical scalability.

In contrast, unsupervised methods optimize registration by maximizing image similarity metrics (e.g., mutual information, local cross-correlation), eliminating the need for manual annotations. These methods are particularly suitable for ultrasound images, which often suffer from high noise and low contrast. Nevertheless, such approaches are prone to local minima during optimization and may fail in scenarios involving significant cardiac motion or deformation.

Weakly supervised learning offers a compromise by introducing sparse annotations—such as anatomical landmarks or segmentation masks—to guide the registration process. This reduces annotation costs while enhancing anatomical plausibility. However, the performance of these methods is highly sensitive to the quality and spatial distribution of the weak labels. Furthermore, their adaptability across modalities remains an open research question.

Traditional medical image registration techniques include feature-based methods (e.g., SIFT, SURF), which establish spatial correspondences through manually engineered features such as anatomical landmarks or edge contours. These approaches depend heavily on the quality of the extracted features and can be negatively affected by ultrasound speckle noise or partial volume effects in CT. Intensity-based methods [42] (e.g., mutual information, the Demons algorithm) directly exploit grayscale information to compute similarity measures. While suitable for multimodal registration, these methods often suffer from low computational efficiency and struggle with large deformations due to their iterative optimization process.

Subsequently, we compare several representative studies that utilize the same dataset. Ameneh et al. [43], Zhang et al. [44], Hering et al. [45], Wang et al. [46], and Chang et al. [47] all employed the ACDC dataset for network training and evaluation in the context of cardiac image registration. Among them, Ameneh et al. adopted a supervised learning approach; Wang et al. and Chang et al. utilized unsupervised methods; while Zhang et al. and Hering et al. implemented weakly supervised frameworks. For reference, the performance of traditional registration methods—SyN and FFD—proposed by Avants et al. [48] and Modat et al. [49], respectively, is also reported on the ACDC dataset in terms of Dice similarity coefficient.

According to the results summarized in Table 6, deep learning-based registration methods outperform traditional techniques in terms of accuracy, with the model by Ameneh et al. achieving the best overall performance. Additionally, deep learning approaches exhibit significantly faster processing speeds due to their ability to leverage GPU acceleration for direct transformation estimation. In terms of computational cost and efficiency, deep learning-based methods demonstrate clear advantages over traditional registration techniques.

Despite the remarkable progress of deep learning in cardiac image registration, its translation into clinical practice remains limited. A major barrier lies in the intrinsic cross-modal discrepancies between cardiac CT and ultrasound: CT provides static, high-resolution anatomical reference, whereas ultrasound captures dynamic, operator-dependent, and often noisy sequences. These differences hinder robust feature correspondence under realistic clinical conditions. In catheterization laboratories and intraoperative settings, for instance, the constant cardiac motion, respiratory influence, and probe-induced deformation challenge the stability of learned registration models. Moreover, most existing approaches are validated on retrospective or single-center datasets that do not reflect the diversity of patient anatomies, imaging protocols, or vendor-specific system characteristics. Evaluation is also largely confined to theoretical similarity metrics—such as Dice coefficient or landmark error—that fail to reflect clinical relevance, particularly regarding navigation accuracy and motion tracking.

To advance clinical applicability, future research should emphasize frameworks that adapt to real-world variability and integrate seamlessly into clinical workflows. Semi-supervised and unsupervised learning strategies, coupled with generative models (e.g., diffusion-based data synthesis), could expand multimodal training data and enhance model generalization to unseen clinical conditions. Multi-task learning architectures that jointly perform registration, segmentation, and motion estimation would enable consistent feature representation for both anatomical alignment and functional assessment during interventional procedures. Embedding biomechanical or physiological priors—such as myocardial strain constraints—into deep architectures may improve prediction stability and interpretability for clinicians. Furthermore, clinically driven evaluation standards that quantify registration benefits in terms of navigation precision, interventional safety, and diagnostic outcomes are essential to replace purely mathematical metrics. Finally, the establishment of large-scale, multi-center datasets and standardized acquisition protocols will be critical to validate model robustness and promote the routine adoption of deep learning–based cardiac multimodal registration in clinical environments.

## 5. Conclusions

This review provides a comprehensive review of both traditional and deep learning-based registration techniques for cardiac CT and ultrasound (US) imaging. Traditional registration methods primarily rely on feature matching, intensity similarity metrics, or biomechanical models to estimate rigid or non-rigid transformations. These approaches require stable and consistent feature extraction, which is challenging given the complex deformations of the heart and the significant intensity differences between CT and US images. Deep learning-based medical image registration offers a new paradigm for achieving accurate fusion between cardiac CT and US. Unlike conventional methods based on hand-crafted features or iterative optimization, deep learning enables automated feature extraction and nonlinear deformation prediction through end-to-end network architectures, significantly improving both accuracy and efficiency. The advantages of deep learning-based approaches are threefold: (1) deep networks can adaptively learn complex spatial correspondences between modalities, addressing challenges such as high noise levels in US and varying resolutions in CT; (2) data-driven learning strategies provide strong generalization capabilities, allowing robust performance under cardiac motion and deformation; and (3) by integrating attention mechanisms and anatomical priors, these models better preserve the topological consistency of critical structures such as myocardium and valves, offering a reliable spatial alignment foundation for downstream clinical applications including diagnosis and interventional planning.

## Figures and Tables

**Table 1:** Intensity-Based Image Registration for Cardiac CT and Ultrasound images.

Reference	Method	Transform	Modality	Dice
Rahimi et al. [3]	NMI	Rigid	CT-US/MRI	0.92 ± 0.05
Chieng et al. [11]	NMI + MI + NCC	Non-rigid	CT-US/MRI	-
Dahman et al. [12]	MI	Rigid	CT-US	0.88 ± 0.05
Khalil et al. [13]	NMI	Rigid	CT-US	0.86 ± 0.05
Abiri et al. [14]	NMI	Non-rigid	CT-MRI	0.92 ± 0.05
Oliveira et al. [15]	NMI + MI + SSD	Non-rigid	CT-US/MRI	-

**Table 6:** Dice coefficient values of different methods on the ACDC dataset.

	Reference	Dice	Time
Deep learning-based registration methods.	Ameneh et al. [43]	0.92	0.07 s (GPU)
	Wang et al. [46]	0.802	5.026 s (GPU)
	Chang et al. [47]	0.833	0.3 s (GPU)
	Zhang et al. [44]	0.859	—
	Hering et al. [45]	0.865	0.006 s (GPU)
Traditional registration methods	Avants et al. [48]	0.702	0.86 s (CPU)
	Modat et al. [49]	0.751	2.23 s (CPU)

## Data Availability

Data and materials will be made available on request. In order to avoid the disclosure of patients’ privacy, relevant original data are not disclosed to the public.

## List of Acronyms

Abbreviations	Description
NMI	Normalized Mutual Information
NCC	Normalized Cross-Correlation
MI	Mutual Information
SSD	Sum of Squared Differences
SIFT	Scale–Invariant Feature Transform
ICP	Iterative Closest Point
HAMMER	Hierarchical Attribute–Matching Mechanism for Efficient Registration
3D ResNet	3D Residual Network
CNN	Convolutional Neural Network
MLP	Multi-Layer Perceptron
SURF	Speeded-Up Robust Features
B-spline	Basis Spline
MS-DIRNet	Multi-scale deformable network
FBA DLIR	Feedback Attention Deep Learning Image Registration
HRF	Hybrid registration framework
STN	Spatial Transformer Network
LSTM	Long Short-Term Memory
